# Pursuing the efficient operation of the primary healthcare hospitals in Thailand through efficiency assessment using the data envelopment analysis method

**DOI:** 10.1017/S1463423624000513

**Published:** 2024-10-29

**Authors:** Siripattra Juthamanee, Aimutcha Wattanaburanon, Yuvadee Rodjarkpai, Saowanee Thongnopakun, Winai Puttakul

**Affiliations:** 1 Faculty of Public Health, Burapha University, Chonburi, Thailand; 2 Faculty of Economics, Kasetsart University, Bangkok, Thailand

**Keywords:** data envelopment analysis, efficiency, primary healthcare, sub-district or Tambon Health Promoting Hospitals

## Abstract

**Aims:**

This study aimed to assess the operational efficiency of Sub-District or Tambon Health Promoting Hospitals (THPHs) in Thailand’s Eastern Economic Corridor (EEC) and to propose management guidelines for future improvements.

**Background:**

The current state of Thailand’s public health demonstrates that government policy has prioritized equal access to public health services in all areas. This increases the need for primary public health services, yet resources are limited and cannot be increased to meet the growing demand. The only effective way to address this issue is to develop the efficiency of public health operations.

**Methods:**

The sample consisted of 104 THPHs in Chachoengsao, a province in Thailand’s EEC. Data for five input and seven output variables were collected between September 18 and November 15, 2023. An online survey was conducted to gather the required data for fiscal year 2022. Data envelopment analysis was used to measure the efficiency of THPHs.

**Findings:**

The average efficiency index of the 104 THPHs was 0.9066, with about 60% having an efficiency index of 1.00. When classified by size, it was found that the efficiency levels of the THPHs grew with size, considering that the average efficiency index of the small, medium, and large THPHs was 0.8642, 0.9140, and 0.9417, respectively. The proportion of efficient THPHs also increased with size, at 58.14%, 60.00%, and 66.67%, respectively. Regarding efficiency improvement targets, small THPHs had the highest output targets (28.40%), followed by medium THPHs (15.31%) and large THPHs (9.91%). For the inefficient THPHs, some management guidelines were made to improve their future performances.

## Introduction

The current global public health situation reveals that an increasing demand for public health services, both in quantity and quality, has led to an exponential growth in resource demand. However, most public health agencies have limited resources and cannot keep up with the growing demand for public health services (Pinprathip, 2018; Jithitikulchai, 2020; Kuzior et al., 2022). It appears that the only approach to increasing public health services with existing resources is to improve operational efficiency (Cylus et al., 2016; Trakakis et al., 2022).

The acceleration of policies to enhance the Thai public health system’s efficiency was evident in 2001, when Thailand changed its public health service provision from a fee-for-service model, where patients bear all of the costs, making it difficult for many low-income people to have access to public health services, to a managed care system, with the goal of ensuring universal access to public health services (Strategy and Planning Division, Ministry of Public Health, 2002). This resulted in public health agencies having inadequate resources to provide services to target groups and to respond to increasing public health needs. The application of the managed care system reflects the vital role of primary healthcare (PHC) services in preventing illness at the grassroots level through the use of various health promotion and disease prevention measures and providing treatment for patients at early stages in order to reduce the number of severe cases, all of which are current responsibilities of PHC agencies. Operational efficiency will help in reducing workloads and in optimizing the use of public health resources for higher-level agencies. Thus, it can be said that the managed care system has made public health agencies at all levels pay attention to the effective use of resources and to set an ultimate goal to become efficiency-oriented.

Sub-district or Tambon Health Promoting Hospitals (THPHs) are the primary public health agencies in Thailand. There are 9,750 THPHs located in sub-district areas nationwide. Thailand’s THPHs can be classified by size, based on the number of their personnel and the size of the population in their catchment areas, into three groups: small, medium, and large (Strategy and Planning Division, Ministry of Public Health, 2022). THPHs have four main missions, comprising health promotion, disease prevention, medical treatment, and health rehabilitation, regarding people at the village level. Most of their budget comes from the universal health coverage programme, which is allocated on a capitation basis. THPHs’ efficiency-oriented operations require an appropriate evaluation system in order to obtain useful information for development planning and continuous operational improvement in terms of input optimization and output management. The present study is intended to initiate a practical method for evaluating the operational efficiency of THPHs, which can serve as a tool to develop ‘efficient hospitals’ with the use of data envelopment analysis (DEA).The DEA evaluation results will be useful in establishing goals and directions for efficiency development and management improvement.

This research was conducted in the Eastern Economic Corridor (EEC) of Thailand, a special economic zone of three provinces: Chonburi, Rayong, and Chachoengsao. The EEC has had a rapid increase in population and this has had an effect on the health service system because the population growth has caused increasing demand for health services, while there are insufficient health service units, limited budgets, shortages of personnel, and service delays (Dardaranonda, 2020; Nantanet et al., 2017), leading to service quality problems exceeding the capacity of existing health service units to handle. Therefore, preparation for operational efficiency in the public health system is necessary. This study aimed to assess the operational efficiency of THPHs in the EEC using the DEA method and to propose management guidelines for efficiency improvement of THPHs in the study area.

## Methods

### Efficiency measurement method

DEA was first used to measure efficiency by Charnes et al. (1978), building on Farrell’s (1957) productive efficiency ideas. The basic model of DEA is fractional programming (FP), which is difficult to compute. Later, the FP model was modified into linear programming (LP), making it easy to calculate efficiency values. DEA LP models can be divided according to production technology criteria into two types: constant returns to scale, known as CCR, and variable returns to scale (VRS), commonly called BCC (Banker et al., 1984). Each type can be further divided based on the orientation of measurement into two categories: input-oriented and output-oriented.

DEA is a method for measuring the technical efficiency (TE) of an organization with no specified form of production function and is considered a nonparametric technique. DEA uses the theories of LP as the basis for determining efficiency index values. The DEA method is appropriate if a decision-making unit (DMU), THPH in this case, has multiple inputs and outputs and if the inputs’ and outputs’ market prices are unavailable or difficult to estimate. Therefore, DEA is suitable for measuring the operational efficiency of government agencies or non-profit organizations (Charnes et al., 1978; Gouveia et al., 2016).

In brief, DEA works by using the inputs and outputs of all DMUs to construct an efficiency frontier of DMUs in the form of a linear combination. A DMU located on the frontier is considered 100% efficient since it produces the maximum outputs using a given level of inputs, while a DMU outside the frontier is deemed inefficient or has fewer than 100% efficiency scores. The efficiency index (ranging from 0 to 1.00) is directly proportional to the DMU’s distance from the efficiency frontier. For any given set of weights, a DMU that produces one-half the outputs that a 100% efficient DMU produces using the same level of inputs is considered to have an efficiency index of 0.5.

Based on the above details, the efficiency index obtained through DEA is regarded as a relative efficiency measure. This is because the efficiency index of an inefficient DMU is determined by comparing its inputs and outputs with those of a specific set of efficient DMUs on the efficiency frontier, which is called a reference set.

In this study, a VRS output-oriented model was used to measure the operational efficiency of THPHs. The model can be written as follows.

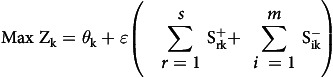

Subject to 
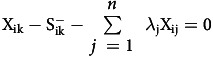
 ; i = 1,2,3,…,m; j = 1,2,3,…,k,…,n


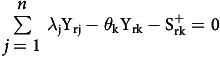
 ; r = 1,2,3,…,s

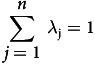





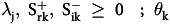
 (unrestricted)


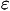
 is an infinitesimally small number,

where

θ_k_ = The output augmentation index of the k^th^ DMU

Y_rj_ = The value of r^th^ output for the j^th^ DMU

X_ij_ = The value of i^th^ input for the j^th^ DMU


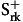
 =The slack variable of the r^th^ output for the k^th^ DMU


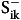
 = The slack variable of the i^th^ input for the k^th^ DMU

The significant data obtained from the application of the chosen DEA model comprised the following: 1) the efficiency index of each THPH (TE), with 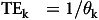 ; 2) the slack variable values of the outputs ( 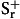 ); and 3) the slack variable values of the inputs ( 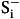 ). These data could be used to determine input and output targets in order to improve the efficiency of inefficient THPHs, as detailed below.

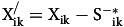




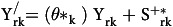

where


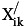
 = The input target value


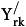
 = The output target value

### Data and variables

EEC consists of three provinces, as indicated, all of which are considered homogeneous in terms of social, economic, and demographic characteristics. Therefore, we defined each province as a cluster and then employed a cluster random sampling approach to select one province, Chachoengsao, as the sample area. Initially, the efficiency of all 117 THPHs in this province was intended to be examined. Data collection was carried out between September 18 and November 15, 2023. Questionnaires were sent online to each THPH to gather THPHs’ inputs and outputs for fiscal year 2022. Follow-up measures were taken in the case of incomplete or delayed data submission. Finally, 104 THPHs provided complete data and served as the sample in this study. A sample size of 104 THPHs highly met the DEA criterion of n ≥ 3(number of input variables + number of output variables) proposed by Banker et al. (1989). The present study had five input variables and seven output variables, so the appropriate sample size should be n ≥ 36.

Selecting variables for use in efficiency evaluation is a critical step. The DEA model’s variables must be able to clearly reflect the operational characteristics of production units, both in terms of inputs and outputs, in order to obtain DEA results that can be used to effectively improve operational efficiency according to organizational contexts (Bowlin, 1998). The researchers selected the variables in this study based on two criteria: 1) they had to be commonly and widely used in PHC performance studies around the world; and 2) they had to be relevant to THPHs’ operational contexts, especially output variables that cover the four main missions. The selection process included two steps: 1) the researchers reviewed relevant literature and research to develop a tentative set of input and output variables and 2) seven experts were asked to examine the content validity using the index of item objective congruence (IOC). Based on the results, five input variables and seven output variables with an IOC value of 0.7 or higher were included in the model. The description of each input variable is provided below.

1. Personnel (I1): Personnel are a significant input variable commonly used in efficiency studies of public health agencies (Pelone et al., 2015; Stefko et al., 2018; Zakowska and Godycki-Cwirko, 2020). There are two types of personnel involved in THPHs’ healthcare service operations: first, THPH employees, and second, village health volunteers (VHVs). Since VHVs are working based on their willingness to help in the tasks of THPHs and THPHs have no direct administrative control over their performances, we decided to employ VHVs as a different input variable (I5).In this study, personnel refers to the total actual number of full-time public health personnel working in THPHs during fiscal year 2022, comprising public health technical officers, registered nurses, Thai traditional medicine doctors, public health officers, and dental assistants. Some THPHs might not have enough personnel for all of the aforementioned positions, depending on their size and the Ministry of Public Health’s manpower framework.
2. Overtime costs (I2): Overall, most THPHs have problems with insufficient numbers of personnel, so they assign personnel to take turns working overtime (OT). In this study, OT costs refer to the total cost for the year 2022 based on reimbursement documents.
3. Pharmaceuticals and medical supplies (I3): THPHs provide public health services to outpatients only. Most of their medical treatment is based on medication and giving accurate health advice, including prenatal care, postpartum care, vaccination services, health screening, mental healthcare, disabled and elderly care, and providing basic medical procedures for non-serious accidents by nurses and public health officers. The measure for I3 is the total expenses incurred during the year 2022 for drugs and medical supplies involving the above-mentioned services as well as medicines for non-communicable diseases (NCDs).
4. Other costs (I4): This input variable covers utility and fuel costs, maintenance costs, and costs for contractual tasks such as cleaning and waste collection.
5. VHVs (I5): VHVs have been established in Thailand since 1977 to play an important role as change agents concerning the health behaviour of local people. Their responsibilities to support and facilitate the work of public health officers, including THPHs, contribute to the efficiency and effectiveness of public healthcare missions. Those that want to be VHVs must have their names listed in the house registration, have lived in the village for at least 6 months and be voluntarily willing to help in public health operations. A group of 10–15 households can vote for one VHV. Once selected, VHVs will receive training according to the curriculum of the Ministry of Public Health and are responsible for taking care of the households in the village (Thianjuruwatthana et al., 2021). In this study, we use the total number of VHVs in each THPH catchment area as the measure of the VHV variable.

The details of the seven output variables are summarized below.

1. Visits and consultations (O1): This output variable is concerned with the number of hospital visits that directly receive medical and public health services in terms of health promotion, disease prevention, disease diagnosis, medical treatment, health rehabilitation, and health consultations. Since THPHs do not have full-time doctors, nurses and public health officers are responsible for providing medical services and useful advice to patients. Doctors from community hospitals or district hospitals in the same PHC network come to see patients at the THPHs from time to time, usually once a month.
2. Health screening (O2): Health screening is an effective approach to health promotion and disease prevention in the THPH healthcare system. It helps to minimize disease incidence, reduce deaths from various diseases, and make people have a better quality of life (Phiphitaporn and Buaphat, 2021). Early disease detection helps patients receive appropriate care in time and can lessen disease severity. The present study uses as the measure of this variable the total number of times that each THPH similarly provides health screening services to general people and high-risk groups to detect common diseases, including diabetes, high blood pressure, cervical cancer, mental disorders, and elderly health status.
3. Quality antenatal care (O3): It is the mission of THPHs to provide health promotion and disease prevention services to pregnant women and infants in order to ensure safe pregnancy and childbirth and to prevent complications or dangers that may occur. Several studies have found that investing in human capital during the first trimester of pregnancy yields a greater economic return than investing at later periods (Doyle et al., 2009). In this study, quality antenatal care refers to services offered to normal pregnant women, including health screening and risk assessment. THPHs should provide health knowledge to pregnant women based on determined standards, provide them with physical examinations, laboratory tests, and essential vitamins, and arrange five doctor or nurse visits for them beginning when the gestational age is ≥ 12 weeks. Pregnant women should also receive continuous care from public health agencies until after giving birth. The measure of this variable is the total number of pregnant women receiving quality antenatal care during the year 2022.
4. Postpartum care (O4): This variable refers to the number of postpartum women receiving three health visits by medical and public health personnel and/or VHVs in accordance with the required criteria; the first visit being within seven days of birth, the second within eight to fifteen days, and the third within sixteen to forty-two days.
5. Vaccinations (O5): The mission of THPHs to enhance local people’s immunity through vaccinations is crucial because it can contribute to disease prevention and basic health promotion and minimize patient care expenses. In this study, vaccinations are measured according to the number of times that they are administered to people of all age groups, according to Ministry of Public Health policy.
6. Community healthcare projects (O6): Most of the THPHs’ community healthcare projects obtain most of financial support from the Community Health Security Fund, which receives partial funding from the National Health Security Fund to encourage local public health units, THPHs, and other related local agencies, groups, and clubs to submit budget requests for new healthcare projects necessary for the target groups in their communities, including pregnant women, postpartum women, toddlers, preschoolers, students, adolescents, working-age people, the elderly, chronically ill people, disabled people, and NCD patients. Most of the projects of the THPHs receiving a budget from this fund are initiated to support the THPHs’ main missions. (National Health Security Office, 2018).This output measure is the total number of projects, both ongoing and completed, during fiscal year 2022.
7. Referrals (O7): In this study, referral refers to the number of times that patients were referred from THPHs to higher-level hospitals or public health agencies. As THPHs are PHC hospitals, there may be limitations in terms of resources or specialized personnel for treating complex illnesses. As a result, THPHs have a mission to coordinate with other public health agencies to refer patients in the community to receive appropriate medical services.

## Results

### Descriptive statistics

The five input variables in this study encompass all of the resources that THPHs need in order to produce their output. Two of them are human resources, while the remaining three involve financial resources required for THPHs’ operations. Meanwhile, the seven output variables were chosen to reflect the four missions of THPHs (health promotion, disease prevention, disease treatment, and health restoration). Table 1 shows the maximum, minimum, average, and standard deviation values of each variable categorized by THPHs’ size.

**Table 1.** Descriptive statistics of input and output variables

Input variables							Output variables								
Size	Value	I1	I2	I3	I4	I5	Size	Value	O1	O2	O3	O4	O5	O6	O7
S (n= 43)	Max	7.00	461,780.00	309,510.00	420,162.00	102.00	S	Max	27,693.00	11,376.00	19.00	16.00	2091.00	6.00	72.00
	Min	2.00	126,000.00	117,600.00	94,773.00	24.00		Min	3,750.00	1,834.00	4.00	3.00	214.00	1.00	3.00
	Average	3.86	275,035.57	118,832.16	233,811.58	46.22		Average	8,362.32	4,651.13	7.21	7.69	513.76	2.99	11.68
	S.D.	1.23	217,234.73	97,184.77	158,602.04	16.02		S.D.	5,445.85	1,497.50	3.73	4.09	402.67	2.32	13.57
M (n= 55)	Max	10.00	646,408.00	782,198.00	697,174.00	176.00	M	Max	91,323.00	19,719.00	53.00	49.00	3,280.00	7.00	80.00
	Min	3.00	197,531.00	225,600.00	160,215.00	30.00		Min	9,478.00	3,386.00	10.00	9.00	412	1.00	5.00
	Average	5.27	305,716.23	263,853.48	399,876.83	75.75		Average	15,439.58	7,976.23	19.36	21.53	798.21	2.89	21.91
	S.D.	1.73	207,407.42	156,343.62	265,103.71	27.76		S.D.	14,839.23	2,930.94	9.84	11.43	820.96	1.80	27.11
L (n= 6)	Max	11.00	789,080.00	789,166.00	687,415.00	250.00	L	Max	112,103.00	28,327.00	73.00	58.00	3,361.00	6.00	68.00
	Min	6.00	295,930.00	304,243.00	181,284.00	112.00		Min	15,404.00	8689.00	18.00	12.00	430.00	2.00	11.00
	Average	7.19	401,358.71	411,132.42	376,588.69	138.65		Average	19,035.21	13,001.09	35.07	32.79	661.14	3.13	25.07
	S.D.	1.37	146,572.93	216,212.89	167,853.04	59.82		S.D.	6,420.36	4,453.66	22.26	19.70	476.88	1.03	20.31

I1 = personnel, I2 = overtime costs, I3 = pharmaceuticals and medical supplies, I4 = other costs, I5 = village health volunteers.

O1 = visits and consultations, O2 = health screening, O3 = quality antenatal care, O4 = postpartum care, O5 = vaccinations.

O6 = community healthcare projects, O7 = referrals.

According to the universal coverage policy, the Thai government establishes networks of PHC agencies, mostly at the district level. Each network consists of one secondary or tertiary hospital, called the Contracting Unit for Primary Care (CUP), and a number of PHC agencies, including THPHs, all of which are located in the vicinity of the CUP. The CUP, or network hospital, has primary responsibilities that include receiving capitation budgets from the National Health Security Fund and distributing them to the network members, handling patient referrals, and providing academic support. Also, the CUP works hand in hand with the District Public Health Office, a policy unit, to facilitate cooperation, resource sharing, and experience exchange in both management and operations among network members (National Health Security Office, 2021). There are 12 CUP networks in Chachoengsao province.

### Efficiency index of THPHs

The efficiency index of each THPH was determined using the VRS output-oriented DEA model. The online DEA software (http://www.onlineoutput.com/dea-software) was applied for calculation. Table 2 shows the efficiency index classified by the THPHs’ size. It was found that the overall operational efficiency of THPHs in Chachoengsao, based on fiscal year 2022 data, was at a high level of about 90%. The average efficiency index was reported at 0.9066. Of all the 104 THPHs, 62 (59.62%) had an efficiency index of 1.00 or achieved 100% efficiency in producing current outputs from existing resources. When classifying the THPHs into three size groups, it was found that the efficiency of the THPHs increased with size, considering that the average efficiency index of small, medium, and large THPHs was 0.8642, 0.9140, and 0.9417, respectively. The proportion of efficient THPHs also increased with size at 58.14%, 60.00%, and 66.67%, respectively. Moreover, the minimum efficiency index of small, medium, and large THPHs had a direct variation with size at 0.4608, 0.6005, and 0.6620, respectively. The influence of organizational size on the operational efficiency of THPHs will be discussed in the following section.

**Table 2.** Efficiency index, classified by size of THPHs

Size of THPHs	Number of THPHs	Number of efficient THPHs (%)	Number of inefficient THPHs (%)	Efficiency index			
				Max	Min	Average	S.D.
Small	43	25 (58.14)	18 (41.86)	1.00	0.4608	0.8642	0.1824
Medium	55	33 (60.00)	22 (40.00)	1.00	0.6005	0.9140	0.1316
Large	6	4 (66.67)	2 (33.33)	1.00	0.6620	0.9417	0.1380
Total	104	62 (59.62)	42 (40.38)	1.00	0.4608	0.9066	–

### Input and output targets for efficiency improvement of inefficient THPHs

Evaluating efficiency using the DEA method not only reveals how well each agency can use existing resources to produce outputs but also provides useful information in setting input and output targets for development planning and management improvement. The obtained targets can be used to boost the efficiency of inefficient THPHs with an efficiency index less than 1.00 based on their potential. This study applied the output-oriented model, which focuses on achieving output maximization with given inputs. According to the principles of LP, the basic DEA technique for calculating the efficiency index, efficiency improvement means adjusting the level of output to a target position on the efficiency frontier. Thus, the output-oriented model used in this study places importance on improving THPH outputs rather than input minimization. Consequently, based on the input and output targets shown in Table 3, in order to improve the efficiency of inefficient THPHs, the increase in outputs in all groups of hospitals must be higher than the decrease in inputs. Small THPHs must increase all types of outputs by an average of 28.40% and decrease all types of inputs by 7.19%. Further, medium THPHs must increase outputs by an average of 15.31% and decrease inputs by 6.65%; and large THPHs must increase outputs by an average of 9.91% and decrease inputs by 5.27%. It should be noted that the percentage of input and output improvement targets is highest in small THPHs, followed by medium and larger THPHs, reflecting the different efficiency levels of each group. As small THPHs have the lowest average efficiency index (0.8642), they require the highest level of improvement. Medium THPHs have the second-lowest average efficiency index (0.9140), and large THPHs with the highest average efficiency index (0.9417) need the lowest level of improvement.

**Table 3.** Input and output targets, classified by size of inefficient THPHs

Size Variables	Small (n=18)			Medium (n=22)			Large (n=2)		
	Average of the original	Average of the target	Change (%)	Average of the original	Average of the target	Change (%)	Average of the original	Average of the target	Change (%)
Input									
I1	4.05	3.73	−7.90	5.67	5.34	−5.82	7.33	7.13	−2.73
I2	293,014.44	251,596.81	−14.14	323,145.45	296,646.36	−8.20	419,204.00	419,203.97	−0.01
I3	137,714.13	134,183.57	−2.56	279,735.48	274,953.75	−1.71	428,067.19	428,067.19	0.00
I4	280,060.48	255,143.63	−8.90	422,050.81	360,564.30	−14.57	385,547.29	365,196.33	−5.28
I5	50.05	48.82	−2.46	81.87	79.44	−2.97	151.50	123.75	−18.32
Average	–	–	−7.19	–	–	−6.65	–	–	−5.27
Output									
O1	7,476.21	9,258.09	23.83	14,423.91	16,142.53	11.92	17,604.00	18,997.96	7.92
O2	3,814.14	4,721.91	23.80	7,152.27	7,973.14	11.48	12,448.05	13,252.17	6.46
O3	6.86	8.78	27.99	17.60	19.56	11.14	32.50	33.69	3.66
O4	7.08	9.29	31.21	18.62	21.42	15.04	30.33	31.35	3.36
O5	497.95	643.54	29.24	788.91	924.42	17.18	640.17	784.25	22.51
O6	2.88	3.63	26.04	2.71	3.14	15.87	2.33	2.50	7.30
O7	10.96	14.98	36.68	20.91	26.04	24.53	22.67	26.79	18.17
Average	–	–	28.40	–	–	15.31	–	–	9.91

### Reference set as benchmarks for the improvement of inefficient THPHs

A reference set is another useful piece of data obtained through the DEA efficiency measurement. It refers to 100% efficient THPHs (TE = 1.00) on the efficiency frontier and functions as a benchmark for the improvement of inefficient THPHs. Each inefficient THPH has a different reference set; for example, in Table 4, THPH15 has two efficient THPHs (THPH20 and THPH91) as its reference set, while THPH92 and THPH82 have five and eight efficient THPHs as their reference sets, respectively. From the management perspective, efficient THPHs in the reference sets are regarded as benchmarks whose best practices can be applied to improve the efficiency of inefficient THPHs. Moreover, for efficiency improvement at the provincial level, there should be further study to gather and analyse data regarding the best practices of various efficient THPHs in order to gain insights to formulate management guidelines for improving the operational efficiency of inefficient THPHs in the province.

**Table 4.** Reference sets for 3 examples of inefficient THPHs

Inefficient THPH	Efficiency index	Reference set							
THPH15	0.9025	THPH20	THPH91						
THPH92	0.8019	THPH2	THPH7	THPH17	THPH33	THPH55			
THPH82	0.6172	THPH21	THPH29	THPH33	THPH34	THPH36	THPH39	THPH80	THPH84

## Discussion and recommendations

The research results revealed that larger THPHs outperformed smaller THPHs, both in terms of the average efficiency index and related figures. The explanation for this outperformance is involved with workload division as well as the output quantity and the quality of each employee in the THPH. Normally, each THPH will assign the four main missions to its employees. Since large THPHs have a large number of employees, they have a sufficient-size task force with expertise and specific skills to fulfil all of their missions and to produce a wide range of high-quality outputs. On the other hand, small THPHs have an insufficient number of skilled employees to complete their four missions, so each employee must be in charge of several tasks, possibly leading to the inability to fulfil all of the organization’s set output goals. In short, as larger THPHs can accomplish all missions and produce many outputs of high quality, they have higher operational efficiency than smaller THPHs, which are unable to successfully perform their missions and create satisfactory outputs. The results of this study indicate the need to prioritize and improve the efficiency of small THPHs in all aspects, including appropriate resource allocation, personnel competency development, and continuous supervision and monitoring.

The DEA output-oriented efficiency measurement model used in this study focuses on enhancing operational efficiency by maximizing outputs with existing inputs. The findings of this study indicate that by increasing all outputs to an average moderate level, we may raise the efficiency of inefficient THPHs and reach the objective of 100% efficiency. As a result, the next step is to develop strategies to boost outputs and hence improve the efficiency of inefficient THPHs. Learning from the best practices or benchmarks of efficient THPHs is an appropriate approach, as mentioned in the reference set section; however, it is outside the scope of this research. Thus, the researchers attempted to formulate some plausible management guidelines for increasing THPH outputs and efficiency based on the information gathered from the present research findings, academic and professional perspectives, and related research as follows.

1. Applying technology to operations: Modern health technology should be applied to THPH operations, such as telemedicine, which can solve the problem of medical personnel shortages in remote areas. The telemedicine system enables medical personnel working in network hospitals to communicate with patients in THPHs through information technology and multimedia equipment, increasing THPHs’ capacity for the organization’s output expansion, including healthcare consultations (O1) in various aspects such as disease prevention, health promotion, and health rehabilitation, while also facilitating convenient and timely medical services regardless of doctor-patient distance (Huncharoen et al., 2014; Sudsom et al., 2023). Currently, telemedicine has been applied in some localities, concentrating on providing health services to chronically ill patients, increasing patient access to medical services, and reducing congestion in public health agencies (Wongprakornkul, 2020).
2. Providing knowledge: Numerous studies have found that people’s lack of knowledge is a major reason causing them not to receive the public health services that are necessary for their personal and family’s well-being, (Yothawut, 2019; Pinnark, 2020; Harnsomboon, 2022). Giving people knowledge could potentially lead to increasing almost all THPH outputs, most apparently visits and consultations (O1), health screening (O2), quality antenatal (O3), postpartum care (O4), and vaccinations (O5). Thus, organizing activities to thoroughly educate each target group about health issues and healthcare practices will increase people’s health awareness, understanding, and willingness to receive public health services from THPHs. Providing knowledge to the general public can be done in various ways, such as organizing training, publishing brochures, posting on social media (e.g., the LINE application), broadcasting on community radio, using community leaders and VHVs as mediums, and holding exhibitions.
3. Proactive services: Bringing public health services to people in the village or community is regarded as a close-to-home, close-to-the-heart strategy (Chaokhonchai and Jamklang, 2022), which helps to increase people’s access to public health services and reduce their concerns about the inconvenience, lack of time, and costs that are caused by public health agencies located far from them (Kankhwao et al., 2023). Many of THPHs’ duties can be completed through proactive service practices and consequently would help to increase organization’s outputs, such as health screening (O2), postpartum care (O4), and vaccinations (O5). THPHs’ personnel can schedule a date and time to provide proactive services and select a place that is at the centre of the community, such as a community hall, temple, or community ground, making it convenient for people to join. All target groups should be thoroughly informed about these proactive services and related information, including the benefits of the universal health insurance card covering free health screening (Kraikaew et al., 2020).
4. The community participation approach to working: All THPHs in Thailand work based on community participation, particularly for projects. THPHs allow people to participate in all stages of a community health project so that they can identify problems behind the project and define implementation procedures and expected benefits together. This approach will give people a sense of belonging and make them willing to collaborate until the project is completed. THPHs can increase the number of community health projects (O6) that meet the needs of local people by conducting a community meeting in each village within their catchment areas so that local people can brainstorm ideas about major health issues for each group of community members (National Health Security Office, 2018) and then develop a project to request a budget from the Community Health Security Fund. The working group for each project mostly consists of people that volunteer to work for the community, while public health officers serve as consultants, monitoring and ensuring that everything proceeds as planned.

Lastly, regarding patient referral (O7), which is a public health mission involving coordination efforts, it is important to make sure that patients with illnesses exceeding the capacity of THPHs can be referred to receive proper treatment at network hospitals (e.g., community hospitals). Therefore, in providing referral services, THPHs prioritize quality over quantity. In other words, THPHs must prepare referral documents, coordinate with other hospitals, and advise patients and families on what to do during the referral process and how to receive treatment at the destination hospital in a timely and efficient manner.

In addition, this DEA study does not focus on reducing inputs to improve organizational efficiency because it is difficult to implement this approach in Thailand’s primary healthcare system, where a lack of resources remains a long-standing issue. However, there are some management practices that can be used to optimize resource utilization in a worthwhile way: 1) adopting lean management principles (Napathorn, 2018) such as establishing best practices for saving electricity, water, fuel, and paper; 2) using inventory management for economical use of resources such as medicines, medical materials, and office supplies (Makpiboon and Krichanchai, 2022); and 3) improving OT management such as switching from performing OT three hours a day to once on the weekend (e.g., Saturday) in order to reduce weekly OT hours and costs to some extent.

## Conclusion

In Thailand’s current public health system, primary healthcare hospitals continue to face challenges regarding operational efficiency. An appropriate tool must be used to evaluate efficiency in order to yield accurate results that are beneficial for formulating effective public health plans and determining strategies to improve THPHs’ operational efficiency according to the local context and develop THPHs to become an efficient organization that maximizes quality services to meet the healthcare needs of local people, using its full capacity of available resources.
